# Hepatitis C Treatment Uptake among Patients Who Have Received Opioid Substitution Treatment: A Population-Based Study

**DOI:** 10.1371/journal.pone.0166451

**Published:** 2016-11-15

**Authors:** Håvard Midgard, Jørgen G. Bramness, Svetlana Skurtveit, John W. Haukeland, Olav Dalgard

**Affiliations:** 1 Department of Infectious Diseases, Akershus University Hospital, Lørenskog, Norway; 2 Institute of Clinical Medicine, University of Oslo, Oslo, Norway; 3 Norwegian Centre for Addiction Research, University of Oslo, Oslo, Norway; 4 Department of Pharmacoepidemiology, The Norwegian Institute of Public Health, Oslo, Norway; 5 Department of Gastroenterology, Oslo University Hospital, Oslo, Norway; Centers for Disease Control and Prevention, UNITED STATES

## Abstract

**Background and Aims:**

There is limited data on hepatitis C (HCV) treatment uptake among people who inject drugs including individuals receiving opioid substitution treatment (OST). We aimed to calculate cumulative HCV treatment uptake, estimate annual treatment rates, and identify factors associated with HCV treatment among individuals who have received OST in Norway.

**Methods:**

This observational study was based on linked data from The Norwegian Prescription Database and The Norwegian Surveillance System for Communicable Diseases between 2004 and 2013. Both registries have national coverage. From a total of 9919 individuals who had been dispensed OST (methadone, buprenorphine or buprenorphine-naloxone), we included 3755 individuals who had been notified with HCV infection. In this population, dispensions of HCV treatment (pegylated interferon and ribavirin), benzodiazepines, selective serotonin reuptake inhibitors and antipsychotics were studied.

**Results:**

Among 3755 OST patients notified with HCV infection, 539 (14%) had received HCV treatment during the study period. Annual HCV treatment rates during OST ranged between 1.3% (95% confidence interval [CI] 0.7–2.2) in 2005 and 2.6% (95% CI 1.9–3.5) in 2008 with no significant changes over time. HCV treatment uptake was not associated with age or gender, but associated with duration of active OST (adjusted odds ratio [aOR] 1.11 per year; 95% CI 1.07–1.15), high (> 80%) OST continuity (aOR 1.62; 95% CI 1.17–2.25), and heavy benzodiazepine use (aOR 0.65; 95% CI 0.49–0.87).

**Conclusions:**

Cumulative HCV treatment uptake among OST patients notified with HCV infection in Norway between 2004 and 2013 was 14%. Annual treatment rates during OST remained unchanged below 3% per year. High continuity of OST over time and absence of heavy benzodiazepine use predicted HCV treatment uptake. Increased awareness for HCV among OST patients is needed as tolerable and efficient directly acting antiviral treatment is being introduced.

## Introduction

In high-income countries, transmission of hepatitis C virus (HCV) infection mainly occurs among people who inject drugs (PWID) [1]. While the global prevalence of anti-HCV is estimated at 1–2% [2], the majority of countries report anti-HCV prevalence estimates above 60% among PWID [1]. The burden of HCV-related liver disease in this population is increasing, particularly among older individuals [3–5]. Although HCV treatment for PWID has shown good outcomes [6, 7] and is recommended by international guidelines [8–11], treatment uptake has remained low in community-based cohorts of PWID (< 2% per year) [12–15] as well as in the general population (< 5% per year in most countries) [16, 17]. This can be attributed to a number of treatment barriers, most notably the lack of suitable models of care and concerns of potential psychiatric adverse effects of interferon (IFN)-based treatment [18–20]. However, with increasing use of tolerable and highly effective IFN-free directly acting antiviral (DAA) regimens, HCV treatment for PWID should become more feasible.

Opioid substitution treatment (OST) could play a key role in the management of the HCV epidemic among PWID [21]. Modelling studies have shown that scaling up HCV treatment combined with improved coverage of OST and needle and syringe programs can prevent onwards transmission and lead to substantial reductions in HCV prevalence [22, 23]. Furthermore, OST programs could provide a platform for linkage to HCV care using existing frameworks for multidisciplinary addiction care. HCV assessment and treatment within such integrated models has shown its feasibility in several studies [24–33]. However, HCV treatment uptake among OST patients has to our knowledge not been documented at the population level.

Currently, the population of PWID in Norway comprises about 15 000 individuals, of whom 50% are receiving OST [34–36]. The prevalence of chronic HCV in this population is approximately 50% and has been stable during the last decade [26, 36, 37]. However, one third of Norwegian OST patients still inject drugs [36] and may therefore continue to be at risk of HCV exposure or contribute to onwards transmission. Documenting HCV treatment uptake in this population is crucial to inform epidemiological models, guide health political decisions and monitor treatment rates as new DAA regimens are being introduced. The high quality of the Norwegian Prescription Database, covering all dispensions of prescription drugs nationwide, provides a unique opportunity to pursue this question at the population level in a pharmaco-epidemiological context, documenting the baseline HCV treatment uptake of the IFN-based treatment era.

The primary aim of this study was to calculate cumulative HCV treatment uptake among individuals who have received OST in Norway. The secondary aims were to estimate annual HCV treatment rates and to identify factors associated with HCV treatment.

## Materials and Methods

This is a population-based observational study based on linked data from The Norwegian Prescription Database (NorPD) and The Norwegian Surveillance System for Communicable Diseases (MSIS). The study population comprised all individuals identified in NorPD who had received OST and were notified to MSIS with HCV infection. The study period for both registries was from January 1 2004 to December 31 2013.

### NorPD

NorPD was established in January 2004 and covers the entire Norwegian population (5.1 million inhabitants). The database contains information on all prescription drugs dispensed at pharmacies to individual patients living outside institutions. For each prescription, the date of dispension and detailed drug information is registered. The reason for the prescription is not recorded, but the diagnosis can be evident though reimbursement codes and some drug prescriptions can be used as a proxy for disease. From 2008, OST has been registered also among institutionalized patients, but prior to 2008 some prescriptions of OST have been missed by the registry.

All drugs in Norway are classified according to the Anatomical Therapeutic Chemical (ATC) Classification System. The quantities of dispensed drugs are measured as the number of defined daily doses (DDDs), which is the assumed average dose per day used for its main indication in adults as determined by the World Health Organization Collaborating Centre for Drug Statistics Methodology [38].

### MSIS

HCV infection has been subject to mandatory notification to MSIS from clinicians and laboratories in Norway since 1990. Initially only cases of acute HCV infection were registered, but from 2008 all HCV infection has been registered based on detection of either anti-HCV antibodies or HCV RNA. For each individual, the date and method of detection, mode of transmission, place of residence and country of origin are registered. However, MSIS is limited by vulnerable notification routines relying on paper forms instead of of a robust automatic electronic system. Experience shows that paper forms often are not completed, and an important proportion of HCV infections therefore probably remain un-notified. Furthermore, the registry does not discriminate well between chronic HCV infection and acute HCV infection with spontaneous clearance.

### Study setting

The Norwegian OST model was implemented in 1998 and is operated through regional specialized centres. Initially the program had a high threshold for admission [39], but new national guidelines from 2010 [40] stated opioid dependency as the only absolute criterion. HCV testing activity in OST programs has generally been low with considerable regional variation [36], and routines for linkage to specialist care have not been systematically implemented.

During the study period, HCV treatment in Norway was delivered by specialist health care services and had not been formally integrated in OST programs, general practice or low threshold settings. Although no longer regarded as an absolute criterion, six months of abstinence from injecting drug use has often been required before consideration for HCV treatment [41]. In Norway, HCV genotype 3 is most common (50%), followed by genotype 1 (40%), genotype 2 (5%) and genotypes 4, 5 and 6 (5%) [42]. Except for individuals with genotype 1 infection, treatment has been offered regardless of the stage of liver fibrosis [43]. During the study period, all treatment regimens were based on a combination of pegylated IFN alpha and ribavirin for 12 to 48 weeks, but from 2014, new DAA treatment has become available in Norway [43]. There is universal health coverage in Norway. Until 2016, HCV treatment was reimbursed by Social Security, and currently, the cost of DAAs are covered by the Regional Health Authorities.

### Study population

OST patients were identified in NorPD by including all individuals who had been dispensed methadone mixture (ATC code N07BC02), buprenorphine sublingual tablets (N07BC01) or buprenorphine–naloxone combined sublingual tablets (N07BC51) at least once during the study period. However, some off-label prescribing of these drugs to patients with chronic pain conditions or cancer does happen. Thus, individuals receiving methadone tablets or capsules more frequently than methadone mixture were excluded because these formulations are primarily used in pain therapy in Norway. In addition, those who received methadone with reimbursements codes for palliative therapy or malignancies and individuals with age below 18 years or above 70 years were excluded.

**Fig 1:**
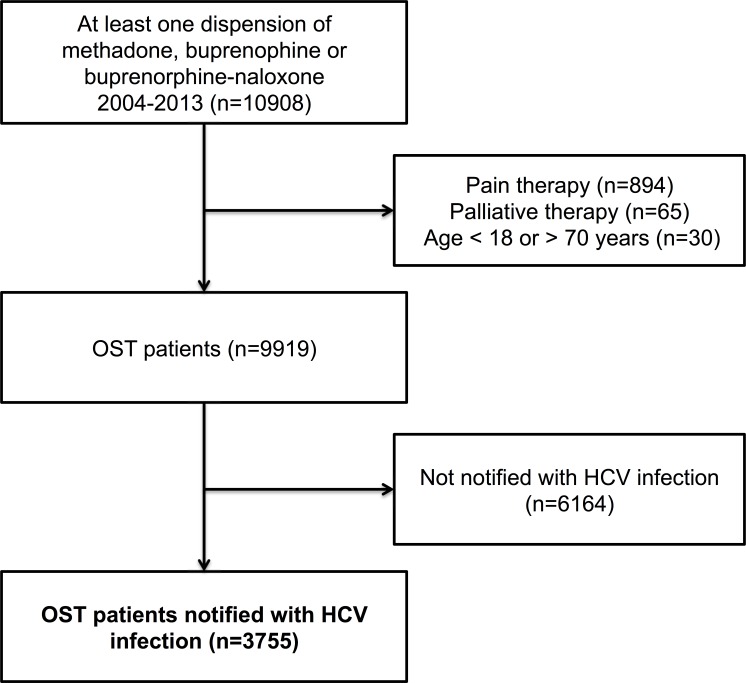
Study population (n = 3755).

The study population was defined as all OST patients identified in NorPD who had been notified to MSIS with HCV infection, regardless of the method of detection was an anti-HCV or HCV RNA test. Fig 1 shows a flow chart defining the study population.

### Measurements

Exposure to HCV treatment was defined as having received ribavirin (J05AB04) and pegylated IFN alpha (L03AB10 or L03AB11) at least once during the study period. The time of HCV treatment was defined by the first registered dispension of ribavirin.

For each participant, duration of active OST was calculated as the total number of months with actual dispensions of OST. OST continuity was defined as the duration of active OST divided by the number of months between first and last dispension (i.e. actual/potential OST duration). Patients were stratified according to OST continuity categories (< 50%, 50–80% or > 80%). Individuals with only one dispension of OST were considered non-adherent and included in the < 50% category. As it has been shown that most dropouts from OST occur within the first year [44], those who had received OST continuously for at least twelve months were also identified.

Dispensions and DDDs of all benzodiazepines (oxazepam, diazepam, nitrazepam, clonazepam, alprazolam and flunitrazepam [N05BA, N05CD and N03AE]) were evaluated. Annual doses were calculated as the total DDDs divided by the time of observation. Patients were stratified according to the following categories of benzodiazepine use: no dispensions, moderate use (≤ mean dose) or heavy use (> mean dose). Finally, dispensions of selective serotonin reuptake inhibitors (SSRIs) (escitalopram, sertraline, citalopram, paroxetine, fluoxetine and fluvoxamine [N06AB]) and antipsychotics (chlorprothixene, quetiapine, olanzapine, levomepromazine, risperidone, aripiprazole, perphenazine, zuclopenthixol, flupenthixol, clozapine, haloperidol, amilsulpride, prochlorperazine, ziprasidone, paliperidone, sertindole and asenapine [N05A]) were studied.

### Statistical analysis

Cumulative HCV treatment uptake was defined as the proportion of individuals who had been dispensed HCV treatment at some point during the study period. Cumulative treatment uptake was stratified according to age groups at end of observation (defined as December 31 2013 or death, whatever came first). Annual HCV treatment uptake was assessed among all individuals who had been dispensed OST in each calendar year of the study period. It was calculated as the annual number of individuals treated for HCV during OST divided by the annual number of previously untreated individuals. To evaluate potential changes over time, Poisson Exact 95% confidence intervals (CIs) for annual rates were calculated.

Data were summarized using frequency (n) and percentage (%) or mean and standard deviation (SD). Factors associated with HCV treatment were evaluated using logistic regression analysis. Unadjusted and adjusted odds ratios (OR) with corresponding 95% CI and *p* values were calculated. Potential predictors were determined a priori and included the following variables: gender (male vs. female), age at initiation of OST (years), country of origin (Norwegian vs. non-Norwegian), duration of active OST (years), OST continuity (50–80% and > 80% vs. < 50%), OST drug (methadone vs. buprenorphine-based), benzodiazepine use (moderate use and heavy use vs. none), SSRI use (any dispensions vs. none) and antipsychotics use (any dispensions vs. none). Uncorrelated variables significant at the 0.10 level in univariate analysis were considered for multivariate analysis and removed with a stepwise elimination approach until only factors significant at the 0.05 level remained in the model. All analyses were performed using the Statistical Package for the Social Sciences (SPSS) version 22.

### Data handling and ethics

The regional committee for ethics in medical research in Norway (REK) approved the study. The study was conducted according to the principles expressed in the Declaration of Helsinki. Data from MSIS were linked to NorPD based on the personal identity number assigned to each person in Norway at birth or immigration. The linkage was performed by a third party (Statistics Norway) as required by the Norwegian law for national health registries, and data was presented in encrypted files providing the confidentiality required. Data on residency were not permitted due to small sample sizes in certain regions. Informed consent from the participants was not collected as all data were analysed anonymously.

## Results

### Characteristics of the study population

From a total of 9919 OST patients identified in NorPD, 3755 (38%) individuals who had been notified to MSIS with HCV infection were included as the study population. The study population increased from 791 individuals in 2004 to 2923 individuals in 2013, with a substantial number of individuals entering and leaving throughout, including 219 (6%) individuals who died. The mean age at initiation of OST was 36 years, 70% were male and 95% had Norwegian origin (Table 1). The mean duration of active OST was 3.8 years and the majority (77%) had received buprenorphine-based OST (Table 1).

**Table 1:** Characteristics of the study population (n = 3755) and factors associated with hepatitis C treatment.

Variable	Overall	Treated for HCV	Not treated for HCV	Unadjusted OR (95% CI)	*p*	Adjusted OR (95% CI)	*p*
Total participants, n (%)	3755 (100)	539 (14)	3216 (86)	-	-	-	-
Age at initiation of OST (years), mean (SD)	36 (9)	36 (8)	36 (9)	1.00 (0.99–1.01)	.935	-	-
Age at end of observation (years), mean (SD)	42 (9)	43 (9)	42 (9)	1.01 (1.00–1.02)	.093	-	-
Age at end of observation, n (%)							
< 40 years	1614 (43)	222 (41)	1392 (43)	1.00	-	-	-
40–49 years	1344 (36)	198 (37)	1146 (36)	1.08 (0.88–1.33)	.448	-	-
≥ 50 years	797 (21)	119 (22)	678 (21)	1.10 (0.87–1.40)	.436	-	-
Gender, n (%)							
Female	1133 (30)	153 (28)	980 (31)	1.00	-	-	-
Male	2622 (70)	386 (72)	2236 (70)	1.11 (0.90–1.35)	.329	-	-
Ethnic origin, n (%)^a^							
Non-Norwegian	193 (5)	19 (4)	174 (6)	1.00	-	-	-
Norwegian	3520 (95)	515 (96)	3005 (95)	1.57 (0.97–2.54)	.067	-	-
Duration of active OST (years), mean (SD)	3.8 (2.7)	4.6 (2.8)	3.6 (2.7)	1.13 (1.10–1.17)	< .001	1.11 (1.07–1.15)	< .001
OST drug, n (%)							
Buprenorphine-based	2904 (77)	411 (76)	2493 (78)	1.00	-	-	-
Methadone only	851 (23)	128 (24)	723 (23)	1.07 (0.87–1.33)	.516	-	-
OST continuity (%), mean (SD)	76 (25)	81 (22)	76 (25)	-	< .001^c^	-	-
OST continuity, n (%)							
< 50%	631 (17)	52 (10)	579 (18)	1.00	-	1.00	-
50–80%	1002 (27)	134 (25)	868 (27)	1.72 (1.23–2.41)	.002	1.38 (0.98–1.95)	.069
> 80%	2122 (57)	353 (66)	1769 (55)	2.22 (1.64–3.02)	< .001	1.62 (1.17–2.25)	.004
OST dropout, n (%)							
Dropout within 12 months	1534 (41)	162 (30)	1372 (43)	1.00	-	-	-
Continuous OST > 12 months	2221 (59)	377 (70)	1844 (57)	1.73 (1.42–2.11)	< .001	-	-
Benzodiazepine dose^b^ mean (SD)	225 (393)	161 (280)	236 (408)	-	< .001^c^	-	-
Benzodiazepine use, n (%)							
No dispensions	574 (15)	97 (18)	477 (15)	1.00	-	1.00	-
Moderate use (< mean dose)	2068 (55)	318 (59)	1750 (54)	0.89 (0.70–1.15)	.376	0.96 (0.75–1.24)	.746
Heavy use (> mean dose)	1113 (30)	124 (23)	989 (31)	0.62 (0.46–0.82)	.001	0.65 (0.49–0.87)	.004
SSRI dose^b^, mean (SD)	29 (87)	35 (90)	28 (86)	-	< .001^c^	-	-
SSRI use (overall), n (%)							
No dispensions	2365 (63)	302 (56)	2063 (64)	1.00	-	-	-
At least one dispension	1390 (37)	237 (44)	1153 (36)	1.40 (1.17–1.69)	< .001	-	-
SSRI use (prior to HCV treatment), n (%)							
No dispensions	2428 (65)	365 (68)	2063 (64)	1.00	-	-	-
At least one dispension	1327 (35)	174 (32)	1153 (36)	0.85 (0.70–1.04)	.109	-	-
Antipsychotics dose^b^, mean (SD)	25 (82)	22 (75)	26 (84)	-	.382^c^	-	-
Antipsychotic use, n (%)							
No dispensions	1693 (45)	254 (47)	1439 (45)	1.00	-	-	-
At least one dispension	2062 (55)	285 (53)	1777 (55)	0.91 (0.76–1.09)	.304	-	-

HCV, hepatitis C virus; OST, opioid substitution treatment; SSRI, selective serotonin reuptake inhibitor; SD, standard deviation; OR, odds ratio; CI, confidence interval

^a^ Missing data for 42 individuals

^b^ Defined Daily Doses (DDDs) per year

^c^ Independent-samples T-test

### Treatment uptake

**Fig 2:**
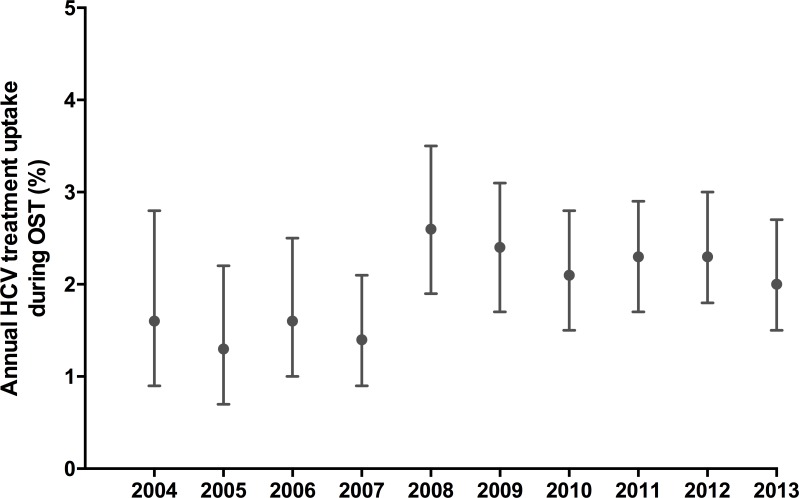
Annual hepatitis C treatment uptake during opioid substitution treatment (n = 3755). Dots indicate estimated treatment rates and bars represent 95% confidence intervals.

Of 3755 HCV infected individuals included as the study population, 539 (14%) had received HCV treatment during the study period. Of those, 111 (21%) were treated before initiation of OST, 375 (70%) were treated during OST, while 53 (10%) were treated after cessation of OST (Table 2). When stratified by age groups at end of observation, cumulative HCV treatment uptake was 14% (222 of 1614), 15% (198 of 1344) and 15% (119 of 797) among individuals aged < 40, 40–49 and ≥ 50 years, respectively. Annual HCV treatment uptake during OST ranged between 1.3% (95% CI 0.7–2.2) in 2005 and 2.6% (95% CI 1.9–3.5) in 2008 (Table 2). Despite overlapping CIs, rates for 2004–2007 trended to be lower than rates for 2008–2013 (Fig 2).

**Table 2:** Annual hepatitis C treatment uptake among individuals who received opioid substitution treatment and were notified with hepatitis C virus infection between 2004 and 2013 (n = 3755).

	2004	2005	2006	2007	2008	2009	2010	2011	2012	2013	Total
Treated for HCV, n											
Prior to OST	20	17	13	18	17	8	9	6	1	2	111
During OST	13	15	22	22	45	46	46	55	59	52	375
After OST	0	0	0	2	3	5	6	7	12	18	53
Total	33	32	35	42	65	59	61	68	72	72	539
Study population, n											
Total	791	1153	1385	1633	1820	2082	2392	2699	2882	2923	-
Untreated	791	1137	1356	1571	1736	1952	2195	2437	2558	2552	-
Treatment uptake during OST, %	1.6	1.3	1.6	1.4	2.6	2.4	2.1	2.3	2.3	2.0	-
95% CI	0.9–2.8	0.7–2.2	1.0–2.5	0.9–2.1	1.9–3.5	1.7–3.1	1.5–2.8	1.7–2.9	1.8–3.0	1.5–2.7	-

HCV, hepatitis C virus; OST opioid substitution treatment; CI, confidence interval

### Individuals excluded from the study population

**Fig 3:**
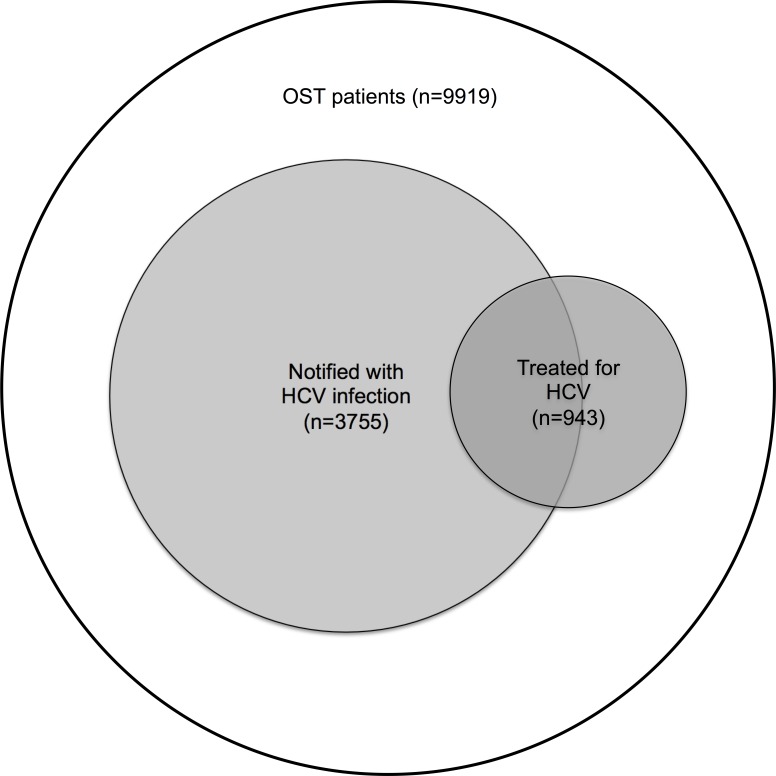
Venn diagram illustrating the relationship between all opioid substitution treatment (OST) patients (n = 9919), OST patients notified with hepatitis C virus (HCV) infection (study population; n = 3755) and all OST patients treated for HCV infection (n = 943). Of all OST patients, 38% were notified with HCV infection. Of all patients treated for HCV infection, 57% were notified with HCV infection.

A substantial proportion of HCV infections are not notified to MSIS, excluding a potentially important number of HCV-infected individuals from the study population. Fig 3 illustrates the relationship between all OST patients (n = 9919), OST patients notified with HCV infection (n = 3755) and OST patients treated for HCV infection (n = 943). Looking at all OST patients identified in NorPD, 943 of 9919 (9.5%) had received HCV treatment during the study period. Thus, 539 of 943 (57%) treated individuals had been notified to MSIS with HCV infection; however, notification rates among treated individuals increased from 39% in 2004–2007 to 76% in 2011–2013. Of 404 of un-notified patients treated for HCV, 223 (55%) had received treatment between 2004 and 2007. Characteristics of notified and un-notified individuals were similar among all OST patients (n = 9919; S1 Table) and among OST patients treated for HCV (n = 943; S2 Table), with the exception that un-notified individuals were older and had higher mortality than notified individuals.

### Factors associated with HCV treatment

Individuals of the study population who had received HCV treatment (n = 539) were compared to those who had not received treatment (n = 3216). In univariate analysis (Table 1), there were no differences in age or gender, but treated individuals had longer duration of active OST, higher OST continuity and less often dropped out of OST within the first year. Patients treated for HCV received lower mean doses of benzodiazepines, were less often heavy benzodiazepine users and were more often dispensed SSRIs. However, the difference in SSRI use was not present when considering SSRIs initiated prior to HCV treatment. There were no differences in dispensions of antipsychotics.

In multivariate analysis (Table 1), HCV treatment was associated with duration of active OST (adjusted OR 1.11 per year; 95% CI 1.07–1.15) and > 80% OST continuity (adjusted OR 1.62; 95% CI 1.17–2.25). Heavy benzodiazepine use was associated with decreased odds (adjusted OR 0.65; 95% CI 0.49–0.87) of receiving HCV treatment. HCV treatment was not associated with age or gender.

## Discussion

This population-based observational study evaluated HCV treatment uptake in Norway between 2004 and 2013 among individuals who had received OST and were notified with HCV infection. Cumulative HCV treatment uptake was 14% and annual treatment rates during OST ranged between 1.3% and 2.6% with no significant changes over time. HCV treatment was associated with duration of active OST, high OST continuity and absence of heavy benzodiazepine use, but was not associated with age or gender. This study provides unique baseline data on HCV treatment uptake among OST patients over a ten-year period prior to the availability of DAA treatment.

The results from this study are consistent with findings from a Norwegian cohort of PWID who previously had been admitted for residential drug dependency treatment, in which 19% of individuals with chronic HCV infection had received HCV treatment during a 16 years observation period [45]. The majority of treated individuals in the present study had received HCV treatment during OST. Still, annual treatment uptake during OST was only marginally higher than treatment rates reported in community-based cohorts of PWID not engaged in OST [12–15]. Although the Norwegian OST program was expanding during the study period, this did not translate into increasing HCV treatment uptake. This could be explained by a reluctance to offer OST patients IFN-based treatment, but might also suggest a low awareness of HCV infection in OST programs in general. Prescription of OST to more vulnerable individuals during the final part of the study period could also have played a role. Stable low treatment rates in this population might therefore reflect ongoing drug use as a barrier to HCV care on both patient- and provider-levels [19]. However, there has been a trend in Norway to increasingly provide HCV treatment for active PWID [43].

Cumulative HCV treatment uptake was similar in all age groups and there was no association between age and HCV treatment. Among diseased HCV RNA positive individuals in a large Norwegian cohort of PWID, advanced liver fibrosis or cirrhosis on autopsy was seen in 35% of those who died 25 years or more after exposure to the virus [46]. In the same cohort, liver disease was the cause of death in 30% of deceased individuals above 50 years of age [4]. Given the high burden of HCV-related liver disease reported from this and other ageing cohorts of PWID with untreated HCV infection [47], it is a concern that treatment uptake was only 15% among individuals above 50 years at the end of the observation.

Certain characteristics of OST were associated with HCV treatment. The odds of receiving HCV treatment increased by 11% for every year spent in active OST. OST continuity by itself was also important; in fact, individuals in active OST more than 80% of the time had 64% increased odds of receiving HCV treatment compared to those with low OST continuity. These are novel findings that raise the hypothesis that retention in OST could promote health-seeking behaviour and facilitate HCV treatment.

This study also found associations between specific drug dispensions and HCV treatment. Heavy, but not moderate benzodiazepine use was associated with decreased odds of receiving HCV treatment, a finding that might reflect a psychosocial vulnerability that characterizes a group of OST patients. Benzodiazepine use is common among Norwegian OST patients and is shown to be associated with negative outcomes including poor social functioning and reduced retention in OST programs [48]. Psychiatric disease is a well-known barrier for IFN-based HCV treatment [18], but no association between dispensions of antipsychotics and HCV treatment was found in this study. SSRI use, however, was more common in patients treated for HCV, but this difference could be attributed to SSRI use initiated during or after HCV treatment. This finding might imply that the increased SSRI use was a consequence of psychiatric side effects of IFN-based treatment [49].

The main strength of this study is its population-based approach, providing a large sample of individuals with opiate dependency who had received OST during a ten-year period. A liberal inclusion of individuals with only sporadic or short-term exposure to OST has ensured a study population more representative of Norwegian PWID. This study is the first to document HCV treatment uptake in this essential target group for HCV treatment, providing important baseline data prior to the availability of DAA treatment.

An inherent limitation of this study is the lack of clinical data available from the registries. This may have impeded detection of factors associated with HCV treatment, although novel pharmaco-epidemiological associations have been identified. Another limitation is that OST administered to institutionalized patients was not registered in NorPD prior to 2008. HCV treatment, however, has almost exclusively been initiated in the outpatient setting and has therefore been captured by the registry throughout the study period. Consequently, annual HCV treatment rates during OST may have been underestimated prior to 2008, since some individuals probably have been misclassified as being treated prior to OST. This might explain the lower trend in treatment rates observed in this period. This bias may also have undervalued OST duration and OST continuity in some individuals, but cumulative HCV treatment uptake has not been affected.

The quality of the MSIS data brings important limitations to this study. Firstly, the registry does not adequately discriminate chronic HCV infections from acute HCV infections with spontaneous clearance. Thus, by including all notified individuals regardless of the method of detection, treatment uptake may have been underestimated. Secondly, the low notification rate is a recognized problem that probably reflects vulnerable notification routines and lacking notifications of chronic infection prior to 2008, as well as low testing activity in OST programs. Also, this study may have missed some individuals notified prior to the study period. Nevertheless, this study has shown that only 38% of OST patients were notified with HCV infection and that only 57% of patients treated for HCV were notified. Although notifications rates among treated individuals improved, it is still a concern that one in four individuals treated for HCV remained un-notified towards the end of the study period.

Restricting the study population to individuals notified with HCV infection has limited the sample size and excluded more than 40% of all patients actually treated for HCV. Although most characteristics were similar between notified and un-notified individuals, un-notified patients were on average three years older than notified individuals. This suggests that the linkage to MSIS may have introduced an age-related selection bias, excluding a group of older HCV infected individuals. Treatment uptake in older age groups may therefore have been underestimated. However, this bias has probably not altered the main finding of the study. Cumulative HCV treatment uptake among all OST patients was 9.5%, and assuming 60% HCV RNA prevalence in the ageing OST population [26, 37], this finding would correspond to 16% treatment uptake among all individuals with presumed chronic HCV infection.

The current availability of tolerable, short-duration and highly efficient DAA regimens has led to significant therapeutic optimism with possibilities for broadened treatment uptake and subsequent HCV elimination among PWID [23, 50–52]. Although derived from IFN-based treatment, the findings from this study are highly relevant, providing baseline data on HCV treatment uptake prior to the introduction of DAAs. Collectively, the findings from this study underscore the need for increased awareness for HCV infection in a growing population of PWID including OST patients now being eligible for HCV treatment. The results should inform health political decisions and support improved HCV testing activity and linkage to HCV care among individuals receiving OST. Although treatment uptake is expected to increase, challenges concerning drug pricing and delivery of care will probably remain. Future studies should therefore monitor treatment rates in this population.

In conclusion, this study has shown that HCV treatment uptake among patients who have received OST in Norway was low and stable during the final ten years of the IFN-based treatment era. Although long-term stability in OST might facilitate HCV treatment, the findings from this study highlight the need for improved awareness for HCV infection in this increasingly important target group for HCV treatment.

## Supporting Information

S1 Table Characteristics of individuals who received opioid substitution treatment between 2004 and 2013, stratified according to hepatitis C virus infection notification status in the Norwegian Surveillance System for Communicable Diseases (MSIS) (n = 9919). (DOCX)

S2 Table Characteristics of individuals who received opioid substitution treatment and were treated for hepatitis C virus (HCV) infection between 2004 and 2013 (n = 943), stratified according to HCV notification status in the Norwegian Surveillance System for Communicable Diseases (MSIS). (DOCX)

## References

[1] Nelson PK, Mathers BM, Cowie B, Hagan H, Des Jarlais D, Horyniak D, et al Global epidemiology of hepatitis B and hepatitis C in people who inject drugs: results of systematic reviews. Lancet. 2011;378(9791):571–83. 10.1016/S0140-6736(11)61097-0 21802134 PMC3285467

[2] Gower E, Estes C, Blach S, Razavi-Shearer K, Razavi H. Global epidemiology and genotype distribution of the hepatitis C virus infection. Journal of hepatology. 2014;61(1 Suppl):S45–57. 25086286 10.1016/j.jhep.2014.07.027

[3] Grebely J, Raffa JD, Lai C, Kerr T, Fischer B, Krajden M, et al Impact of hepatitis C virus infection on all-cause and liver-related mortality in a large community-based cohort of inner city residents. Journal of viral hepatitis. 2011;18(1):32–41. 10.1111/j.1365-2893.2010.01279.x 20196806

[4] Kielland KB, Skaug K, Amundsen EJ, Dalgard O. All-cause and liver-related mortality in hepatitis C infected drug users followed for 33 years: a controlled study. Journal of hepatology. 2013;58(1):31–7. 10.1016/j.jhep.2012.08.024 22960427

[5] Hajarizadeh B, Grebely J, Dore GJ. Epidemiology and natural history of HCV infection. Nature reviews Gastroenterology & hepatology. 2013;10(9):553–62. 23817321 10.1038/nrgastro.2013.107

[6] Aspinall EJ, Corson S, Doyle JS, Grebely J, Hutchinson SJ, Dore GJ, et al Treatment of hepatitis C virus infection among people who are actively injecting drugs: a systematic review and meta-analysis. Clinical infectious diseases: an official publication of the Infectious Diseases Society of America. 2013;57 Suppl 2:S80–9. 23884071 10.1093/cid/cit306

[7] Dimova RB, Zeremski M, Jacobson IM, Hagan H, Des Jarlais DC, Talal AH. Determinants of hepatitis C virus treatment completion and efficacy in drug users assessed by meta-analysis. Clinical infectious diseases: an official publication of the Infectious Diseases Society of America. 2013;56(6):806–16. 23223596 10.1093/cid/cis1007 PMC3582354

[8] AASLD-IDSA. AASLD-IDSA Recommendations for Testing, Managing, and Treating Adults Infected With Hepatitis C Virus. Available: http://www.hcvguidelines.org. Accessed 20 June 2016. (Archived by WebCite® at http://www.webcitation.org/6iPAiC0wA). 2015. 10.1002/hep.27950 26111063

[9] Grebely J, Robaeys G, Bruggmann P, Aghemo A, Backmund M, Bruneau J, et al Recommendations for the management of hepatitis C virus infection among people who inject drugs. The International journal on drug policy. 2015;26(10):1028–38. 10.1016/j.drugpo.2015.07.005 26282715 PMC6130980

[10] WHO. Guidelines for the screening, care and treatment of persons with chronic hepatitis C infection. Available: http://www.who.int/hepatitis/publications/hepatitis-c-guidelines-2016/en/. 2016. 27227200

[11] EASL. European Association for the Study of the Liver. EASL Recommendations on Treatment of Hepatitis C 2016. Journal of hepatology. 2016. 10.1016/j.jhep.2022.10.006 36464532

[12] Mehta SH, Genberg BL, Astemborski J, Kavasery R, Kirk GD, Vlahov D, et al Limited uptake of hepatitis C treatment among injection drug users. Journal of community health. 2008;33(3):126–33. 10.1007/s10900-007-9083-3 18165889 PMC3800027

[13] Grebely J, Raffa JD, Lai C, Krajden M, Kerr T, Fischer B, et al Low uptake of treatment for hepatitis C virus infection in a large community-based study of inner city residents. Journal of viral hepatitis. 2009;16(5):352–8. 10.1111/j.1365-2893.2009.01080.x 19226330

[14] Alavi M, Raffa JD, Deans GD, Lai C, Krajden M, Dore GJ, et al Continued low uptake of treatment for hepatitis C virus infection in a large community-based cohort of inner city residents. Liver international: official journal of the International Association for the Study of the Liver. 2014;34(8):1198–206. 24164865 10.1111/liv.12370

[15] Iversen J, Grebely J, Topp L, Wand H, Dore G, Maher L. Uptake of hepatitis C treatment among people who inject drugs attending Needle and Syringe Programs in Australia, 1999–2011. Journal of viral hepatitis. 2014;21(3):198–207. 10.1111/jvh.12129 24438681

[16] Razavi H, Waked I, Sarrazin C, Myers RP, Idilman R, Calinas F, et al The present and future disease burden of hepatitis C virus (HCV) infection with today's treatment paradigm. Journal of viral hepatitis. 2014;21 Suppl 1:34–59. 24713005 10.1111/jvh.12248

[17] Hatzakis A, Chulanov V, Gadano AC, Bergin C, Ben-Ari Z, Mossong J, et al The present and future disease burden of hepatitis C virus (HCV) infections with today's treatment paradigm—volume 2. Journal of viral hepatitis. 2015;22 Suppl 1:26–45. 25560840 10.1111/jvh.12351

[18] McGowan CE, Fried MW. Barriers to hepatitis C treatment. Liver international: official journal of the International Association for the Study of the Liver. 2012;32 Suppl 1:151–6. 22212587 10.1111/j.1478-3231.2011.02706.x PMC3955982

[19] Bruggmann P. Accessing Hepatitis C patients who are difficult to reach: it is time to overcome barriers. Journal of viral hepatitis. 2012;19(12):829–35. 10.1111/jvh.12008 23205675

[20] McGowan CE, Monis A, Bacon BR, Mallolas J, Goncales FL, Goulis I, et al A global view of hepatitis C: physician knowledge, opinions, and perceived barriers to care. Hepatology. 2013;57(4):1325–32. 10.1002/hep.26246 23315914 PMC3683983

[21] Perlman DC, Jordan AE, Uuskula A, Huong DT, Masson CL, Schackman BR, et al An international perspective on using opioid substitution treatment to improve hepatitis C prevention and care for people who inject drugs: Structural barriers and public health potential. The International journal on drug policy. 2015. 10.1016/j.drugpo.2015.04.015 PMC4581906 26050614

[22] Vickerman P, Martin N, Turner K, Hickman M. Can needle and syringe programmes and opiate substitution therapy achieve substantial reductions in hepatitis C virus prevalence? Model projections for different epidemic settings. Addiction. 2012;107(11):1984–95. 10.1111/j.1360-0443.2012.03932.x 22564041

[23] Martin NK, Hickman M, Hutchinson SJ, Goldberg DJ, Vickerman P. Combination interventions to prevent HCV transmission among people who inject drugs: modeling the impact of antiviral treatment, needle and syringe programs, and opiate substitution therapy. Clinical infectious diseases: an official publication of the Infectious Diseases Society of America. 2013;57 Suppl 2:S39–45. 23884064 10.1093/cid/cit296 PMC3722076

[24] Mauss S, Berger F, Goelz J, Jacob B, Schmutz G. A prospective controlled study of interferon-based therapy of chronic hepatitis C in patients on methadone maintenance. Hepatology. 2004;40(1):120–4. 10.1002/hep.20279 15239094

[25] Hallinan R, Byrne A, Agho K, Dore GJ. Referral for chronic hepatitis C treatment from a drug dependency treatment setting. Drug and alcohol dependence. 2007;88(1):49–53. 10.1016/j.drugalcdep.2006.09.018 17067763

[26] Krook AL, Stokka D, Heger B, Nygaard E. Hepatitis C treatment of opioid dependants receiving maintenance treatment: results of a Norwegian pilot study. European addiction research. 2007;13(4):216–21. 10.1159/000104884 17851243

[27] Litwin AH, Harris KA Jr., Nahvi S, Zamor PJ, Soloway IJ, Tenore PL, et al Successful treatment of chronic hepatitis C with pegylated interferon in combination with ribavirin in a methadone maintenance treatment program. Journal of substance abuse treatment. 2009;37(1):32–40. 10.1016/j.jsat.2008.09.009 19038524 PMC2692471

[28] Witteck A, Schmid P, Hensel-Koch K, Thurnheer MC, Bruggmann P, Vernazza P, et al Management of hepatitis C virus (HCV) infection in drug substitution programs. Swiss medical weekly. 2011;141:w13193 10.4414/smw.2011.13193 21623473

[29] Sasadeusz JJ, Dore G, Kronborg I, Barton D, Yoshihara M, Weltman M. Clinical experience with the treatment of hepatitis C infection in patients on opioid pharmacotherapy. Addiction. 2011;106(5):977–84. 10.1111/j.1360-0443.2010.03347.x 21205057

[30] Alavi M, Grebely J, Micallef M, Dunlop AJ, Balcomb AC, Day CA, et al Assessment and treatment of hepatitis C virus infection among people who inject drugs in the opioid substitution setting: ETHOS study. Clinical infectious diseases: an official publication of the Infectious Diseases Society of America. 2013;57 Suppl 2:S62–9. 23884068 10.1093/cid/cit305

[31] Masson CL, Delucchi KL, McKnight C, Hettema J, Khalili M, Min A, et al A randomized trial of a hepatitis care coordination model in methadone maintenance treatment. American journal of public health. 2013;103(10):e81–8. 10.2105/AJPH.2013.301458 23947319 PMC3853128

[32] Bruggmann P, Litwin AH. Models of care for the management of hepatitis C virus among people who inject drugs: one size does not fit all. Clinical infectious diseases: an official publication of the Infectious Diseases Society of America. 2013;57 Suppl 2:S56–61. 23884067 10.1093/cid/cit271 PMC6279207

[33] Grebely J, Alavi M, Micallef M, Dunlop AJ, Balcomb AC, Phung N, et al Treatment for hepatitis C virus infection among people who inject drugs attending opioid substitution treatment and community health clinics: the ETHOS Study. Addiction. 2016;111(2):311–9. 10.1111/add.13197 26451534

[34] Amundsen EJ, Bretteville-Jensen AL. Hard drug use in Norway. Nordic Studies on Alcohol and Drugs. 2010;27.

[35] Skretting A. BEK, Vedøy, T. F., Lund, K. E. Drug use in Norway. SIRUS report 2015. The Norwegian Institute for Alcohol and Drug Research. Available: http://wpstatic.idium.no/www.sirus.no/2015/12/rusmidler_i_norge2015.pdf. Accessed 20 June 2016. (Archived by WebCite® at http://www.webcitation.org/6iPCCbZEJ).

[36] Waal H, Busserud K, Clausen T, Skeie I, Håseth A, Lillevold H. Annual assessment of the Norwegian OMT program 2016. SERAF, University of Oslo. Avaiable: http://www.oslo-universitetssykehus.no/SiteCollectionDocuments/Omoss/Avdelinger/Psykiskhelseogavhengighet/Rus-ogavhengighetsbehandling/NasjonalkompetansetjenesteTSB/seraf-rapport-nr-1-2016-statusrapport-2015.pdf. Accessed 20 June 2016. (Archived by WebCite® at http://www.webcitation.org/6iPCcZGfV).

[37] Dalgard O, Egeland A, Ervik R, Vilimas K, Skaug K, Steen TW. [Risk factors for hepatitis C among injecting drug users in Oslo]. Tidsskrift for den Norske laegeforening: tidsskrift for praktisk medicin, ny raekke. 2009;129(2):101–4. 10.4045/tidsskr.09.35002 19151801

[38] Guidelines for ATC classification and DDD assignment 2013 WHO Collaborating Centre for Drug Statistics Methodology, Norwegian Institute of Public Health, Oslo, Norway Available: http://www.whocc.no. Accessed 20 June 2016. (Archived by WebCite® at http://www.webcitation.org/6iPBKwpRP).

[39] Waal H. Merits and problems in high-threshold methadone maintenance treatment. Evaluation of medication-assisted rehabilitation in Norway 1998–2004. European addiction research. 2007;13(2):66–73. 10.1159/000097935 17356277

[40] National guidelines for opioid substitution treatment 2010. The Norwegian Directorate of Health. Available: https://helsedirektoratet.no/Lists/Publikasjoner/Attachments/100/IS-1701-Legemiddelassistert-rehabilitering-ved-opioidavhengighet.pdf. Accessed 20 June 2016. (Archived by WebCite® at http://www.webcitation.org/6iPB3f7a0).

[41] Bell H, Dalgard O, Bjoro K, Hellum KB, Myrvang B. [Treatment of chronic hepatitis C]. Tidsskrift for den Norske laegeforening: tidsskrift for praktisk medicin, ny raekke. 2002;122(9):926–8. 12082838

[42] Norwegian Institute of Public Health. Available: https://wwwfhino/nettpub/smittevernveilederen/sykdommer-a-a/hepatitt-c—veileder-for-helsepers/-forekomst-i-norge. 2016.

[43] Dalgard O, Bjøro K, Mæland A, Konopski Z, Karlsen L, Sandvei P, et al. Norwegian guidelines for assessment and treatment of hepatitis C infection 2015. Available: http://www.hepatittfag.no. Accessed 20 June 2016. (Archived by WebCite® at http://www.webcitation.org/6iPBWGkvc).

[44] Bukten A, Skurtveit S, Waal H, Clausen T. Factors associated with dropout among patients in opioid maintenance treatment (OMT) and predictors of re-entry. A national registry-based study. Addictive behaviors. 2014;39(10):1504–9. 10.1016/j.addbeh.2014.05.007 24960556

[45] Kielland KB, Amundsen EJ, Dalgard O. HCV treatment uptake in people who have injected drugs—observations in a large cohort that received addiction treatment 1970–1984. Scandinavian journal of gastroenterology. 2014;49(12):1465–72. 10.3109/00365521.2014.968860 25310139

[46] Kielland KB, Delaveris GJ, Rogde S, Eide TJ, Amundsen EJ, Dalgard O. Liver fibrosis progression at autopsy in injecting drug users infected by hepatitis C: a longitudinal long-term cohort study. Journal of hepatology. 2014;60(2):260–6. 10.1016/j.jhep.2013.09.022 24096048

[47] Grebely J, Dore GJ. What is killing people with hepatitis C virus infection? Seminars in liver disease. 2011;31(4):331–9. 10.1055/s-0031-1297922 22189973

[48] Bramness JG, Kornor H. Benzodiazepine prescription for patients in opioid maintenance treatment in Norway. Drug and alcohol dependence. 2007;90(2–3):203–9. 10.1016/j.drugalcdep.2007.03.008 17478058

[49] Sulkowski MS, Cooper C, Hunyady B, Jia J, Ogurtsov P, Peck-Radosavljevic M, et al Management of adverse effects of Peg-IFN and ribavirin therapy for hepatitis C. Nature reviews Gastroenterology & hepatology. 2011;8(4):212–23. 21386812 10.1038/nrgastro.2011.21

[50] Grebely J, Matthews GV, Lloyd AR, Dore GJ. Elimination of hepatitis C virus infection among people who inject drugs through treatment as prevention: feasibility and future requirements. Clinical infectious diseases: an official publication of the Infectious Diseases Society of America. 2013;57(7):1014–20. 23728143 10.1093/cid/cit377

[51] Grebely J, Dore GJ. Can hepatitis C virus infection be eradicated in people who inject drugs? Antiviral research. 2014;104:62–72. 10.1016/j.antiviral.2014.01.002 24468275

[52] Hickman M, De Angelis D, Vickerman P, Hutchinson S, Martin NK. Hepatitis C virus treatment as prevention in people who inject drugs: testing the evidence. Current opinion in infectious diseases. 2015;28(6):576–82. 10.1097/QCO.0000000000000216 26524330 PMC4659818

