# Supplementary material for: Hepatitis C Treatment Uptake among Patients Who Have Received Opioid Substitution Treatment: A Population-Based Study
Source: PLoS One. 2016 Nov 15;11(11):e0166451. doi: 10.1371/journal.pone.0166451 (PMC5112941; doi:10.1371/journal.pone.0166451)
Supplement: S1 Table — (DOCX) [file pone.0166451.s001.docx]

**S1 Table. Characteristics of individuals who received opioid substitution treatment between 2004 and 2013, stratified according to hepatitis C virus infection notification status in the Norwegian Surveillance System for Communicable Diseases (MSIS) (n=9919).**

| Characteristic | Overall  (n=9919) | Notified with HCV infection (n=3755) | Not notified with HCV infection  (n=6164) |
| --- | --- | --- | --- |
| Age at initiation of OST, mean (SD) | 38 (9) | 36 (9) | 39 (9) |
| Age at end of observation, mean (SD) | 43 (9) | 42 (9) | 44 (9) |
| Age at end of observation, n (%)  < 40 years  40-49 years  ≥ 50 years | 3628 (37)  3762 (38)  2529 (26) | 1614 (43)  1344 (36)  797 (21) | 2014 (33)  2418 (39)  1732 (28) |
| Male gender, n (%) | 6969 (70) | 2622 (70) | 4347 (71) |
| Deaths, n (%) | 1001 (10) | 219 (6) | 782 (13) |
| Duration of active OST (years), mean (SD) | 3.9 (2.8) | 3.8 (2.7) | 3.9 (2.9) |
| Buprenorphine based OST, n (%) | 7093 (72) | 2904 (77) | 4189 (68) |
| OST continuity, mean % (SD) | 77 (26) | 76 (25) | 77 (27) |
| OST continuity > 80%, n (%) | 5881 (59) | 2122 (57) | 3759 (61) |
| Continuous treatment > 1 year, n (%) | 5924 (60) | 2221 (59) | 3703 (60) |
| Benzodiazepine dose (DDD/year), mean (SD) | 246 (509) | 225 (393) | 259 (568) |
| Benzodiazepine use, n (%)  No dispensions  Moderate use (< mean dose)  Heavy use (> mean dose) | 1739 (18)  5192 (52)  2988 (30) | 574 (15)  2068 (55)  1113 (30) | 1165 (19)  3124 (51)  1875 (30) |
| At least one dispension of antipsychotics, n (%) | 5152 (52) | 2062 (55) | 3090 (50) |
| At least one dispension of SSRIs, n (%) | 3661 (37) | 1390 (37) | 2271 (37) |

HCV, hepatitis C virus; OST, opioid substitution treatment; SD, standard deviation; DDD, defined daily doses; SSRI, selective serotonin reuptake inhibitor
