# Supplementary material for: Hepatitis C Treatment Uptake among Patients Who Have Received Opioid Substitution Treatment: A Population-Based Study
Source: PLoS One. 2016 Nov 15;11(11):e0166451. doi: 10.1371/journal.pone.0166451 (PMC5112941; doi:10.1371/journal.pone.0166451)
Supplement: S2 Table — (DOCX) [file pone.0166451.s002.docx]

**S2 Table. Characteristics of individuals who received opioid substitution treatment and were treated for hepatitis C virus (HCV) infection between 2004 and 2013 (n=943), stratified according to HCV notification status in the Norwegian Surveillance System for Communicable Diseases (MSIS).**

| Characteristic | Overall  (n=943) | Notified with HCV infection (n=539) | Not notified with HCV infection  (n=404) |
| --- | --- | --- | --- |
| Age at initiation of OST, mean (SD) | 38 (8) | 36 (8) | 39 (8) |
| Age at end of observation, mean (SD) | 44 (9) | 43 (9) | 46 (8) |
| Age at end of observation, n (%)  < 40 years  40-49 years  ≥ 50 years | 312 (33)  378 (40)  253 (27) | 222 (41)  198 (37)  119 (22) | 90 (22)  180 (45)  134 (33) |
| Male gender, n (%) | 669 (71) | 386 (72) | 283 (70) |
| Deaths, n (%) | 57 (6) | 23 (4) | 34 (8) |
| Duration of active OST (years), mean (SD) | 4.8 (2.8) | 4.6 (2.8) | 5.1 (2.9) |
| Buprenorphine based OST, n (%) | 688 (73) | 411 (76) | 277 (69) |
| OST continuity, mean % (SD) | 81 (22) | 81 (22) | 82 (21) |
| OST continuity > 80%, n (%) | 625 (66) | 353 (66) | 272 (67) |
| Continuous treatment > 1 year, n (%) | 675 (72) | 377 (70) | 298 (74) |
| Benzodiazepine dose (DDD/year), mean (SD) | 163 (293) | 161 (281) | 165 (308) |
| Benzodiazepine use, n (%)  No dispensions  Moderate use (< mean dose)  Heavy use (> mean dose) | 181 (19)  551 (58)  211 (22) | 96 (18)  321 (60)  122 (23) | 85 (21)  230 (57)  89 (22) |
| At least one dispension of antipsychotics, n (%) | 463 (49) | 285 (53) | 178 (44) |
| At least one dispension of SSRIs, n (%) | 388 (41) | 237 (44) | 151 (37) |

HCV, hepatitis C virus; OST, opioid substitution treatment; SD, standard deviation; DDD, defined daily doses; SSRI, selective serotonin reuptake inhibitor
